# The utility of brief instruments for depression screening in dialysis patients

**DOI:** 10.1093/ckj/sfae369

**Published:** 2024-11-22

**Authors:** Isabel Vázquez, Adolfo Figueiras, Ángel Salgado-Barreira

**Affiliations:** Department of Clinical Psychology and Psychobiology, Universidade de Santiago de Compostela, Santiago de Compostela, Spain; Department of Public Health, University of Santiago de Compostela, Santiago de Compostela, Spain; Consortium for Biomedical Research in Epidemiology and Public Health, Madrid, Spain; Health Research Institute of Santiago de Compostela, Santiago de Compostela, Spain; Department of Public Health, University of Santiago de Compostela, Santiago de Compostela, Spain; Consortium for Biomedical Research in Epidemiology and Public Health, Madrid, Spain; Health Research Institute of Santiago de Compostela, Santiago de Compostela, Spain

**Keywords:** Beck Depression Inventory, depression, dialysis, hospital anxiety and depression scale, SF-36

## Abstract

**Background:**

Depression is a frequent but often underdiagnosed comorbid disorder in dialysis patients. The Beck Depression Inventory–Second Edition (BDI-II) is a reliable and valid instrument for depression screening but is relatively long for repeated use in clinical practice. The aim of this study was to compare the BDI-II with the shorter questionnaires Beck Depression Inventory–FastScreen (BDI-FS), the depression subscale of the Hospital Anxiety Depression Scale (HADS-D), the Mental Health (MH) scale of the 36-item Short Form Health Survey (SF-36) and two items of the MH (‘So down in the dumps that nothing could cheer you up’ and ‘Downhearted and blue’) to determine the most efficient instruments for screening depressive symptoms in dialysis patients.

**Methods:**

A cross-sectional study was conducted involving patients from 14 health centres undergoing in-centre haemodialysis or peritoneal dialysis. All patients completed the BDI-II, HADS-D and MH scale. The sensitivity, specificity and positive and negative predictive values for each brief instrument were assessed relative to BDI-II ≥16.

**Results:**

Of the 145 patients included in the study (mean age 62 years; 66% male), 24.8% had depressive symptoms (BDI ≥16). The cut-off points with the highest sensitivity and negative predictive value for BDI-FS were ≥3 (91.7% and 96.1%, respectively) and ≥4 (80.6% and 92.4%, respectively) and for the HADS-D these were ≥4 (91.7% and 95.8%, respectively) and ≥5 (83.3% and 92.6%, respectively). The cut-off points for the total MH and the two items (considered separately or together) resulted in lower sensitivity (<80%) and lower negative predictive values (<90%).

**Conclusions:**

Both the BDI-FS and HADS-D are adequate screening tools for depression in the dialysis population. As the BDI-FS is easier to complete and score and enables identification of patients at risk of suicide, it may be the best alternative to the BDI-II for depression screening in dialysis patients in clinical settings.

## Graphical Abstract

**Graphical Abstract:**
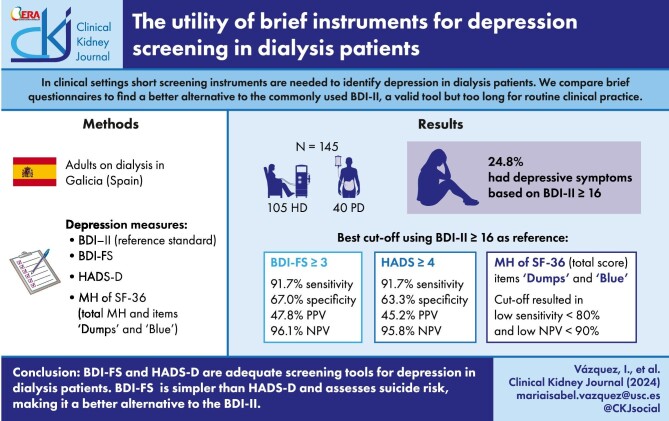

KEY LEARNING POINTS **What was known:**

- Depression is a prevalent mental disorder in dialysis patients but often remains undiagnosed.
- In the routine clinical care of dialysis patients, brief and easy-to-administer screening instruments are needed to identify patients with symptoms of depression, but the most appropriate short screening tool for dialysis patients has not yet been established.

**This study adds:**

- In dialysis patients, the Beck Depression Inventory–Fast Screen (BDI-FS) and the depression subscale of the Hospital Anxiety and Depression Scale (HADS-D) are suitable tools for depression screening with the BDI-FS displaying some advantages over the HADS-D. In contrast, the Mental Health Scale of the 36-item Short Form Health Survey appears to be unsuitable for depression screening.

**Potential impact:**

- The BDI-FS and HADS-D could provide effective, feasible screening for depression in the routine clinical care of dialysis patients, reducing the burden on patients and health staff.
- The BDI-FS exhibits certain advantages over the HADS and may be the best alternative for depression screening in dialysis patients in clinical settings.

## INTRODUCTION

Depression has been described as the most common psychological problem in patients with end-stage renal disease (ESRD) undergoing dialysis treatment [1], with an estimated prevalence of 22.8% when assessed by clinical interview and 39.3% when assessed by self- or clinician-administered questionnaires [2].

Depression or depressive symptoms in renal patients undergoing dialysis is associated with poor health outcomes such as reduced quality of life [3], poor adherence to pharmacological or dietary treatments [4, 5], greater numbers of infections [6, 7], more hospitalizations [8] and higher all-cause mortality [9], including suicide [10].

Despite the high rates of comorbid depression and the additional negative impact on health outcomes in dialysis patients, many patients are undiagnosed and consequently untreated [11, 12]. For many patients, there is a stigma associated with a diagnosis of depression, and affected patients may tend to deny depression-related symptoms [13]. It is therefore important that detection of depression forms part of the care that patients receive.

Structured clinical interview represents the ‘gold standard’ for diagnosing depression [14], but its use in clinical settings has the disadvantages that it must be administered by trained health professionals and is time-consuming.

The Beck Depression Inventory (BDI) is a reliable and valid self-report instrument to assess depressive symptoms and is by far the most widely researched and used questionnaire in patients with renal failure [15]. Its most recent version, the Beck Depression Inventory–Second Edition (BDI-II) [16], complies with the criteria of the *Diagnostic and Statistical Manual of Mental Disorders* [17], and a cut-off point of ≥16 is considered the threshold that best optimizes the balance between sensitivity and specificity in dialysis patients [15, 18]. However, the BDI-II has some important disadvantages for use in renal patients in clinical practice. First, it is a relatively long instrument (21 items), which restricts its repeated use. In addition, it includes somatic symptoms of depression that may mimic symptoms related to the underlying renal diagnosis and treatment (such as fatigue and changes in appetite and/or sleep), which could act as potential confounders for depression screening in dialysis patients [1].

In the routine clinical care of nephrology patients, brief and easy-to-administer screening instruments that do not include physical symptoms are needed to identify patients with symptoms of depression. The Beck Depression Inventory–FastScreen (BDI-FS) [19], the depression scale of the Hospital Anxiety and Depression Scale (HADS-D) [20] and the Mental Health (MH) scale of the 36-item Short Form Health Survey (SF-36) [21] may be useful alternatives to the BDI-II. In dialysis patients, the HADS-D has often been used to assess depressive symptoms [22, 23], and the MH scale or just two MH questions (‘So down in the dumps that nothing could cheer you up’ and ‘Downhearted and blue’) have been used to identify symptoms of depression in dialysis patients in some studies [24–27]. In contrast, the BDI-FS has not been used to assess symptoms of depression in renal patients in epidemiological or intervention studies.

The aim of this study was to compare the BDI-FS, HADS-D and MH (both the total scale and two of its items) to determine which short instruments are the most efficient screening tools and represent the best alternative to the standard, but relatively long, BDI-II for the detection of depressive symptoms in dialysis patients.

## MATERIALS AND METHODS

This cross-sectional study was conducted in ESRD patients undergoing dialysis from 14 hospitals and dialysis centres in Galicia, Spain. Patients were eligible to participate in the study if they were at least 18 years of age, had received the same dialysis modality during the last 3 months and were literate in Spanish. The exclusion criteria were a history of major psychiatric disorders other than depression, unstable clinical state in the last month and active concurrent illness at the time of assessment.

A team of psychologists collected sociodemographic data by interviewing the participants. In addition, the Brief Physical Activity Assessment Tool (BPAAT) [28] was administered to assess the patient's usual physical activity by categorizing the patient as ‘sufficiently active’ (≥3 sessions/week of 20 minutes of vigorous activity or ≥5 sessions/week of 30 minutes of moderate activity or any combination of both) or ‘insufficiently active’ if the above conditions were not met.

The nephrologist responsible for each patient's care collected clinical information and measured comorbidity using the age-adjusted Charlson Comorbidity Index [29].

The psychologists administered in person to each individual patient the following questionnaires:

- BDI-II [16]: a 21-item self-report screening instrument that assesses various degrees of depressive symptomatology. Each item is rated on a 4-point scale ranging from 0 to 3 (maximum total score 63). Higher scores indicate more severe depressive symptoms. On the basis of previous findings, patients with a BDI-II score ≥16 were classified as having depressive symptoms [15, 18].
- BDI-FS [19]: includes seven of the non-somatic symptoms from the BDI-II (sadness, pessimism, fast failure, loss of pleasure, self-dislike, self-criticalness and suicidal thoughts or wishes). The scoring is similar to the BDI-II, with a maximum total score of 21. In this study, the BDI-FS score was calculated from the relevant items of the BDI-II.
- HADS [20]: a 14-item self-report questionnaire in which the anxiety subscale (HADS-A) and the depression subscale (HADS-D) each comprise seven items. Each item is rated on a 4-point Likert scale ranging from 0 to 3 (maximum total score 21). A higher score indicates a higher level of symptomatology.
- MH of the SF-36 [21]: the SF-36 comprises eight scales designed to measure health-related quality of life. The MH scale assesses emotional well-being through five items. Each item is rated on a 6-point scale ranging from 1 to 6. The final MH score is calculated by summing the item scores and transforming this score to a scale ranging from 0 to 100, with lower scores indicating poorer emotional status. In line with previous studies, we also assessed two of the five items of MH as indicators of depressive affect: ‘So down in the dumps that nothing could cheer you up’ (i.e. ‘Dumps’) and ‘Downhearted and blue’ (i.e. ‘Blue’). In this study both the score on each item separately and the sum of both items were considered.

The Spanish adaptations of the questionnaires were used in all cases [30–34]. The order of administration was counterbalanced to account for possible order effects. All questionnaires were administered to patients immediately before or within the first 2 hours of their haemodialysis (HD) treatment and within the waiting time for routine follow-up for peritoneal dialysis (PD) patients.

All participants provided written informed consent. The study was approved by the Galician Ethical Research Committee (reference number 2016/235).

### Statistical analysis

The sociodemographic and clinical characteristics of the sample are presented as frequencies, means, standard deviations (SDs) and range. Cronbach's α was used to determine the reliability of the BDI-II, BDI-FS, HADS-D and MH.

Receiver operating characteristics (ROC) analysis was used to assess the discrimination power of the BDI-FS, HADS-D and MH scale and of the items Dumps and Blue relative to cases defined by the BDI-II (BDI-II ≥16). The discrimination power of the tests was characterized by the area under the ROC curve (AUC), with an AUC ≥0.75 representing good discrimination [35].

Sensitivity, specificity and positive and negative predictive values (PPV and NPV, respectively) were calculated for different cut-off points for each of the instruments studied for cases of depression (BDI-II ≥16). Currently there is no consensus on the performance criteria to be used for depression screening instruments. In light of previous studies evaluating questionnaires used to measure depression in dialysis patients [36, 37], and taking into account the need for high sensitivity, we considered a sensitivity ≥80% as acceptable. We also applied the recommendation that the sum of sensitivity and specificity should be ≥1.5 for a test to be useful [38]. In addition, an NPV ≥90% was considered acceptable, given the importance of reducing the number of false negatives [39]. The best cut-off point associated with the above criteria was then considered to be the threshold. Agreement between the BDI-FS, HAD, MH and two items of the MH and the categorical BDI-II ≥16 was measured using the κ coefficient (values ≥0.40 were considered acceptable [40]).

Data analysis was performed using SPSS Statistics version 29 (IBM, Armonk, NY, USA) and *P*-values <.05 were considered significant.

## RESULTS

A total of 145 patients on maintenance dialysis (72.4% HD and 27.6% PD) were included in the study. The mean age of the entire cohort was 62.3 years and 66.2% were male. The main sociodemographic and clinical characteristics of the sample are shown in Table 1.

**Table 1:** Sociodemographic and clinical characteristics of the sample.

Variables	Values
Age (years), mean ± SD (range)	62.3 ± 14.9 (26–87)
Sex, *n* (%)	
Men	96 (66.2)
Women	49 (33.8)
Marital status, *n* (%)	
Single/divorced/separated/widowed	56 (38.6)
Married or cohabitant	89 (61.4)
Education, *n* (%)	
None/primary	86 (59.3)
Secondary/university	59 (40.7)
Work status, *n* (%)	
Active	6 (4.2)
Disabled	64 (44.8)
Retired	73 (51.0)
Socio-economic level, *n* (%)	
Low/medium-low	44 (30.3)
Medium/medium-high/high	101 (69.7)
Smoking status, *n* (%)	
Non-smoker	65 (45.1)
Ex smoker	62 (4.1)
Smoker	17 (11.8)
Physical activity, *n* (%)	
Sufficiently active	60 (42.9)
Insufficiently active	80 (57.1)
Aetiology of kidney disease, *n* (%)	
Glomerulonephritis	24 (16.7)
Pyelonephritis/Interstitial nephritis	12 (8.3)
Polyschistosis	14 (9.7)
Hereditary/congenital	2 (1.4)
Vascular	11 (7.6)
Systemic	8 (5.6)
Diabetic nephropathy	33 (22.9)
Other	2 (1.4)
Unaffiliated	38 (26.4)
Months in current dialysis modality, mean ± SD (range)	51.4 ± 71.78 (3–504)
Charlson Comorbidity Index adjusted by age, mean ± SD (range)	5.8 ± 2.4 (2–15)
Hospitalizations in the last 6 months, *n* (%)	
None	103 (81.1)
≥1	24 (18.9)
Previous kidney transplants, *n* (%)	
None	95 (74.8)
≥1	32 (25.2)
Haemoglobin (g/dl), mean ± SD (range)	11.5 ± 1.2 (8.3–15.2)
Albumin (g/dl), mean ± SD (range)	3.8 ± 0.3 (3.2–4.7)
Transplant waiting list, *n* (%)	
No	100 (70.4)
Yes	42 (29.6)

All scales examined showed high internal consistency (≥0.70). The highest Cronbach's α values were obtained for the BDI-II (α = 0.88) and the MH scale (α = 0.83) and the values for HADS-D and BDI-FS were somewhat lower (0.77 and 0.75, respectively). At the recommended threshold for the BDI-II (BDI-II ≥16) [15, 18], 36 patients (24.8%) were identified as having depressive symptoms. For the BDI-II, BDI-FS, HADS-D and MH the mean scores were 11.6, 3.2, 4.7 and 73.2, respectively. The mean scores were 5.0 for the Dumps item, 4.6 for the Blue item and 9.6 for the two items considered together (Table 2).

**Table 2:** Reliability and scores for self-reports of depression.

Questionnaire	Reliability	Mean ± SD (range)
BDI-II	0.88	11.6 ± 9.0 (0–47)
BDI-FS	0.75	3.2 ± 3.2 (0–16)
HADS-D	0.77	4.7 ± 4.0 (0–19)
MH	0.83	73.2 ± 20.9 (8 –100)
Item Dumps	NA	5.0 ± 1.2 (1– 6)
Item Blue	NA	4.6 ± 1.3 (1– 6)
Items Dumps and Blue	NA	9.6 ± 2.3 (3–12)

NA: not applicable.

**Figure 1:**
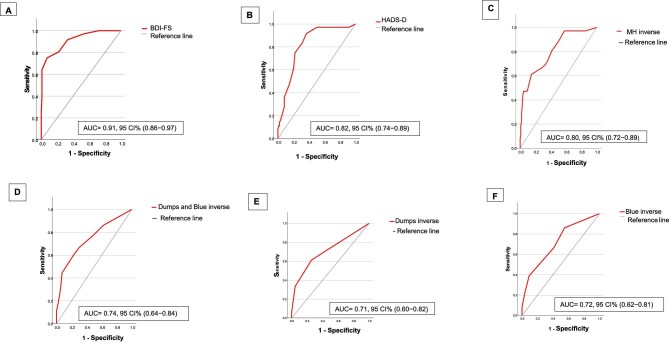
AUCs for **(A)** BDI-FS, **(B)** HADS-D, **(C)** MH of SF-36, **(D)** items Dumps and Blue, **(E)** item Dumps and **(F)** item Blue.

All three questionnaires showed good discrimination of depressive symptoms. The AUC was 0.91 for the BDI-FS, 0.82 for the HADS-D and 0.80 for the MH taking as a reference BDI-II ≥16. The Dumps and Blue items, considered separately or together, had a lower discriminatory power, and the AUC did not exceed the threshold of 0.75 (Fig. 1).

For the BDI-FS, cut-off points of ≥3 and ≥4 were superior to the prespecified threshold for sensitivity, specificity, NPV and κ concordance index. For the cut-off point ≥3, sensitivity (0.917) and NPV (0.961) were higher than for the cut-off point ≥4 (0.806 and 0. 924, respectively), but specificity was lower for the BDI-FS ≥3 (0.670) than for the BDI-FS ≥4 (0.780). The sum of sensitivity and specificity was >1.5 and similar at both cut-off points. Concordance with BDI ≥16 was quite similar for BDI-FS ≥3 (κ = 0.449) and for BDI ≥4 (κ = 0.505).

For the HADS-D, the cut-off points that exceeded the established thresholds were 4 and 5. At the cut-off HADS-D ≥4, the sensitivity was 0.917, the specificity was 0.633 and the NPV was 0.958. Increasing the cut-off (HADS-D ≥5) decreased the sensitivity (0.833) and NPV (0.926), but increased the specificity (0.688). The sum of sensitivity and specificity was similar for both cut-off points and exceeded 1.5. The concordance with BDI ≥16 was similar for both cut-off points (κ = 0.409 and κ = 0.414, respectively).

For the MH and the Dumps and Blue items, considered separately or together, none of the cut-off points met the established criteria for sensitivity, NVP or concordance.

Table 3 summarizes the screening performance of the BDI-FS, HADS-D, MH and the Dumps and Blue items relative to BDI-II ≥16.

**Table 3:** Screening performance of the BDI-FS, HADS-D, MH and items Dumps and Blue using the BDI-II ≥16 to identify depressive symptoms.

Measure	Cut-off score	Sensitivity, % (95% CI)	Specificity, % (95% CI)	PPV, % (95% CI)	NPV, % (95% CI)	Youden Index (95% CI)	κ
BDI-FS	>3	91.7 (81.3–100,0)	67.0 (57.7–76.3)	47.8 (35.3–60.3)	96.1 (91–100)	0.59 (0.46–0.71)	0.449
	>4	80.6 (66.2–94.9)	78.0 (69.7–86.2)	54.7 (40.4–69.1)	92.4 (86.4–98.4)	0.59 (0.43–0.74)	0.505
	>5	75.0 (59.5–90.5)	92.7 (87.3–98.0)	77.1 (61.8–92.5)	91.8 (86.2–97.4)	0.68 (0.53–0.83)	0.683
HADS-D	>4	91.7 (81.3–100.0)	63.3 (53.8–72.8)	45.2 (33.1–57.3)	95.8 (90.5–100)	0.55 (0.42–0.68)	0.409
	>5	83.3 (69.8–96.9)	68.8 (59.7–78.0)	46.9 (33.9–59.9)	92.6 (86.3–98.9)	0.52 (0.37–0.67)	0.414
	>6	75.0 (59.5–90.5)	78.0 (69.7–86.2)	52.9 (38.3–67.6)	90.4 (84.0–96.9)	0.53 (0.37–0.69)	0.465
MH	≤62	61.1 (43.8–78.4)	85.3 (78.2–92.4)	57.9 (40.9–74.9)	86.9 (80.1–93.8)	0.46 (0.29–0.64)	0.456
	≤66	66.7 (49.9–83.5)	71.6 (62.6–80.5)	43.6 (29.6–57.7)	86.7 (79.1–94.3)	0.38 (0.21–0.56)	0.325
	≤70	69.4 (53.0–85.9)	67.0 (57.7–76.3)	41.0 (27.8–54.1)	86.9(79.1–94.7)	0.36 (0.19–0.54)	0.295
	≤74	72.2 (56.2–88.2)	64.2 (54.8–73.7)	40.0 (27.3–52.7)	87.5 (79.6–95.4)	0.36 (0.19–0.54)	0.287
Dumps	≤4	61.1 (43.8–78.4)	74.3 (65.7–83.0)	44.0 (29.2–58.8)	85.3 (77.6–92.9)	0.35 (0.18–0.53)	0.313
Blue	≤4	66.7 (49.9–83.5)	58.7 (49.0–68.4)	34.8 (22.8–46.8)	84.2 (75.4–93.1)	0.25 (0.07–0.43)	0.194
Dumps and Blue	≤8	61.1 (43.8–78.4)	76.2 (67.7–84.6)	45.8 (30.7–61.0)	85.6 (78.1–93.1)	0.37 (0.19–0.55)	0.335
	≤9	66.7 (49.9–83.5)	69.7 (60.6–78.8)	42.1 (28.4–55.8)	86.4 (78.6–94.1)	0.36 (0.19–0.54)	0.304

CI: confidence interval.

## DISCUSSION

Given the high prevalence of depressive symptomatology in the dialysis population and the associated personal and social impact, there is a need to incorporate screening tools in patients with kidney failure as part of their care. A routine screening program for depressive symptoms in dialysis patients must be efficient and cost-effective to be successful. It must be well accepted by ESRD patients and easy to administer by healthcare staff. In the present study, we investigated whether the brief questionnaires BDI-FS, HADS-D and the MH scale (or two items of this scale) represent viable alternatives to the BDI-II, a valid and reliable but more extensive instrument for assessing depressive symptoms in renal patients. Our results suggest that the BDI-FS and HADS-D may be possible alternatives to the BDI-II in clinical practice. The BDI-FS and HADS-D yielded good reliability indices, with α coefficients >0.70 (threshold considered adequate [40]) and AUCs >0.75, which indicates useful discrimination [35].

Since the main aim of our study was to compare the discriminatory power of short questionnaires relative to that of the BDI-II for depression screening in dialysis patients, we chose cut-off scores with high sensitivity and NPV because of their ability to minimize false negatives, even if this leads to lower specificity and PPV and thus a higher probability of false positives. A higher false positive rate in these screening tests may be acceptable given that these questionnaires are inexpensive, easy to administer and do not impose a large burden on patients and health professionals [39]. In addition, and very importantly, the impact of referring dialysis patients for psychiatric interviews at which the diagnosis is not confirmed is likely to be less detrimental than not identifying patients with depressive symptoms who are at risk of poor health outcomes and who are deprived of effective treatment for depression.

According to the established criteria of sensitivity ≥80%, NPV ≥90% and κ coefficient ≥0.40 as acceptable screening accuracy, the performance of BDI-FS cut-off points of 3 and 4 were suitable for identifying patients with depressive symptoms considering BDI-II ≥16 as a reference. These cut-off points displayed good sensitivity (91.7% and 80.6%, respectively) and excellent NPV (96.1% and 92.4%, respectively). Our results are consistent with those reported by Aleshed *et al.* [41] for a cut-off point of 4 (83% sensitivity and 89% NPV) and slightly lower than those obtained by Neitzer *et al.* [42] with a cut-off point of 4 (97.2% sensitivity and 98.9% NPV) [both studies compared the BDI-FS with the BDI-II (BDI-II ≥14 and BDI-II ≥16, respectively)].

On the HADS-D scale, the cut-off points of 4 and 5 met the established criteria for sensitivity (91.7% and 83.3%, respectively) and NPV (95.8% and 92.6%, respectively) and showed acceptable agreement with the BDI-II ≥16 (κ > 0.40 for both cut-off points). In line with previous studies [36, 43], these cut-off points are lower than established for other chronically ill populations (HADS-D ≥8). A wide range of cut-off points for the HADS-D (between 3 and 8) are shown for other chronically ill populations [44], indicating that the most appropriate cut-off point needs to be determined for the patient population being assessed. Our results show that a cut-off point of HADS-D ≥8, despite being the most widely used in dialysis patient studies [45, 46], may not be appropriate for this patient population and is not comparable to a BDI-II score ≥16. Therefore there may be large differences between studies regarding identifying patients with depressive symptoms, the prevalence estimates and/or in assessing the effects of interventions for depression, depending on the instrument and cut-off used.

Although our results suggest that both the BDI-FS and HADS-D are useful instruments for detecting depressive symptoms, the BDI-FS may have some advantages over the HADS-D. On the one hand, the BDI-FS is easier to complete than the HADS-D according to the Fog readability index [47], and the BDI-FS is easier to score since, unlike the HADS-D, the answers to some of the questions do not need to be reversed. On the other hand, the BDI-FS enables identification of patients at risk of suicide [48], which is not covered by the HADS-D. In dialysis patients, high suicide rates have been estimated [49], and it is therefore of interest to have instruments that allow for the evaluation of suicidal ideation without having to include specific measures that increase the burden of routine psychological assessment for patients and healthcare staff.

As regards the MH scale, the SF-36 is frequently administered to dialysis patients, so MH scores are widely available. If this scale proves useful in screening for depressive symptoms in dialysis patients, it would facilitate detection of depressive symptomatology in patients assessed for quality of life without the need to administer additional instruments. This has led to the use of this scale for depression screening in various studies and has been found that the MH score is associated with the risk of cardiovascular events and mortality [24] and severe pruritus [25]. However, in our study, although the MH showed an adequate AUC (0.80) considering the BDI-II as reference, we did not identify a cut-off point that meets the diagnostic performance criteria established in this study.

When considering the MH as a measure of depressive symptoms, it should be borne in mind that the MH was originally designed to assess a more general concept of emotional state (including anxiety, depression, loss of behavioural or emotional control and psychological well-being) [21] rather than specifically depressive symptomatology. In this respect, some studies have considered only the two items of the MH scale that refer specifically to mood (‘So down in the dumps that nothing could cheer you up’ and ‘Downhearted and blue’) as indicators of depressive symptomatology, finding that they were related to mortality [26, 50], increased risk of dialysis withdrawal [50] and increased risk of hospitalization [27]. In the present study, these two items showed low diagnostic accuracy (AUC <0.75), when considered both separately and together. They are therefore not recommended as an alternative to the BDI-II in dialysis patients. Although the emotional distress expressed by patients in the MH scale or in two of its items (Blue and Dumps) does not show a good level of sensitivity to identify cases of patients with clinically relevant depressive symptoms as assessed by the BDI-II, it should be taken into account as an indicator of psychological distress. This psychological distress should be addressed with specific techniques, such as psychoeducational interventions. Such interventions have been shown to decrease psychological distress and improve the health-related quality of life of patients undergoing dialysis [51].

Regarding screening for depression in dialysis patients, the Controversies Conference on Symptom-Based Complications in Dialysis, held by Kidney Disease: Improving Global Outcomes in 2022, reported that given the benefits of identifying depression, including the potential benefits of pharmacological and non-pharmacological treatments, it is recommended that depression screening be offered to all dialysis patients using a validated tool with a defined cut-off point to indicate potential depression in this population [52]. In addition, questionnaires need to be short and easy to administer so that they can be incorporated into the routine clinical care of nephrology patients.

Some studies have also considered brief screening tools for depressive symptoms in dialysis patients. For example, Collister *et al.* [53] proposed a single question from the Edmonton Symptom Assessment System, and Dano *et al.* [37] established a two-step screening procedure using a Patient Health Questionairre-2 score ≥1 followed by a Patient-Reported Outcomes Measurement Information System depression score ≥53. However, it remains to be determined which instrument or procedure is the optimal screening tool for dialysis patients in clinical settings.

Our results suggest that the brief questionnaires BDI-FS and HADS-D are both useful instruments for systematic screening of depressive symptoms in dialysis patients in clinical settings. Following the screening process, those patients with BDI-FS ≥3 or HADS-D ≥4 should be referred to a psychiatrist or clinical psychologist for diagnosis. If indicated, these patients would then receive pharmacotherapy and/or psychological treatment for depression.

The strengths of our study are that it included a sample of dialysis patients selected from a large number of centres, providing a diverse sample of patients and a good reflection of the in-centre HD and PD populations seen in clinical practice in Spain.

Our study also has several limitations that should be taken into account when interpreting the results. First, we used the BDI-II as the reference rather than structured diagnostic interview, which is the gold standard for the diagnosis of depression. Although the BDI-II has high sensitivity and specificity relative to the structured diagnostic interview [18], it is a surrogate marker (although validated), but it is not the gold standard for diagnosing depression. Consequently, the results of this study refer to the identification brief instruments that could be used as alternatives to the BDI-II to identify patients with depressive symptoms, but not to diagnose patients with depression (which must be done by clinical interview). However, it is important to emphasize the significance of identifying depressive symptoms in patients with kidney disease, as they are associated with poor health outcomes [3–6, 8–10], as previously mentioned. Another limitation of the study is that the sample consisted of only Spanish dialysis patients and it is not possible to extrapolate our results to dialysis patients from other countries or cultures or to patients with chronic kidney disease not receiving dialysis treatment or to kidney transplant recipients.

In conclusion, our results show that the BDI-FS and HADS-D are good measures for screening for depressive symptoms in dialysis patients and can be used in routine clinical practice as an alternative to the BDI-II. If these questionnaires are to be used in the dialysis population, it is important to note that the thresholds in both scales need to be modified in dialysis patients, as the recommended cut-off scores are lower than those suggested for a chronically ill population. The BDI-FS and HADS-D seem to be of comparable clinical utility to the BDI-II and have the advantage of requiring less time to answer, which would result in them being easier for patients to complete and for clinicians to score and interpret. This would allow systematic screening for depression in dialysis patients. Although the BDI-FS has scarcely been used in the renal patient population, it has some advantages over the HADS-D and may therefore be the best alternative to the BDI-II for depression screening in dialysis patients in the clinical setting. Further research on the characteristics and usefulness of this instrument in the renal disease population is warranted.

## Data Availability

The data underlying this article will be shared upon reasonable request to the corresponding author.
